# Viral and host proteins involved in picornavirus life cycle

**DOI:** 10.1186/1423-0127-16-103

**Published:** 2009-11-20

**Authors:** Jing-Yi Lin, Tzu-Chun Chen, Kuo-Feng Weng, Shih-Cheng Chang, Li-Lien Chen, Shin-Ru Shih

**Affiliations:** 1 Research Center for Emerging Viral Infections, Chang Gung University, Tao-Yuan, Taiwan; 2 Department of Medical Biotechnology and Laboratory Science, Chang Gung University, Tao-Yuan, Taiwan; 3 Graduate Program in Biomedical Science, Chang Gung University, Tao-Yuan, Taiwan; 4 Division of Biotechnology and Pharmaceutical Research, National Health Research Institutes, Zhunan, Taiwan

## Abstract

Picornaviruses cause several diseases, not only in humans but also in various animal hosts. For instance, human enteroviruses can cause hand-foot-and-mouth disease, herpangina, myocarditis, acute flaccid paralysis, acute hemorrhagic conjunctivitis, severe neurological complications, including brainstem encephalitis, meningitis and poliomyelitis, and even death. The interaction between the virus and the host is important for viral replication, virulence and pathogenicity. This article reviews studies of the functions of viral and host factors that are involved in the life cycle of picornavirus. The interactions of viral capsid proteins with host cell receptors is discussed first, and the mechanisms by which the viral and host cell factors are involved in viral replication, viral translation and the switch from translation to RNA replication are then addressed. Understanding how cellular proteins interact with viral RNA or viral proteins, as well as the roles of each in viral infection, will provide insights for the design of novel antiviral agents based on these interactions.

## Introduction

**Figure 1:**
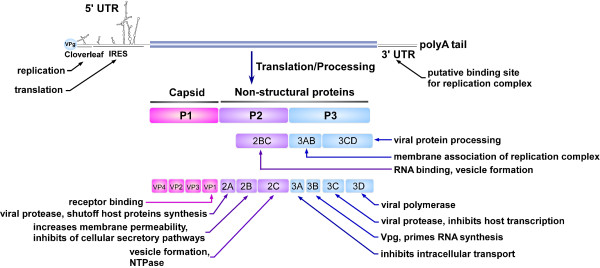
**Schematic of the enterovirus genome, the polyprotein products and their major functions**. A diagrammatic representation of the enterovirus genome is shown. The 11 mature polypeptides are shown, together with the three main cleavage intermediates. The main biological functions are included for each polypeptide. UTR, untranslated region; IRES, internal ribosome entry site; VPg, viral protein genome-linked.

Picornaviruses are a large family of animal viruses, which are pervasive in nature. Certain members of this family are well known since they importantly affect human health. The family *Picornaviridae *consists of five genera - enteroviruses, rhinoviruses, cardioviruses, aphthoviruses, and hepatoviruses. Picornaviruses are small icosahedral particles containing a single-stranded plus sense RNA genome with approximately 7,500 nt in length. It contains a 3' poly(A) tail with a variable length from 65 to 100 nt. The viron RNA has a virus-encoded peptide, VPg, attached at its 5' terminus, but this protein is rapidly lost in the cell and most of the viral transcripts consequently lack it [1,2]. The picornavirus RNAs lack the cap structure (m7GpppN, where m is a methyl group and N is any nucleotide). The viral RNA encodes a single large polyprotein which undergoes a series of processing events, mediated by virus-encoded protease, to produce the mature virus proteins (including 11 mature proteins plus numerous partially processed products, depending on the virus). Four of these proteins (VP1-VP4) constitute the virus capsid, and the others participate in virus replication [3] (Fig. 1).

The infection of cells by enterovirus is an efficient and productive event. To complete the life cycle of the virus, viral proteins are involved in viral replication and translation, in addition to altering host functions, such as cellular gene expression, protein localization, signal transduction and membrane rearrangement. This review focuses on the functions of viral and host factors involved in the life cycle of picornaviruses.

### Host factors and capsid proteins involved in receptor binding

The capsid proteins of picornaviruses are encoded by the P1 region of the genome, and the capsid particles comprise 60 copies of four P1-encoded polypeptides, VP1 to VP4. The first three viral proteins (VP1-VP3) reside on the outer surface of the virus, and the shorter VP4 is located completely on the inner surface of the capsids. The capsid proteins mediate the initiation of infection by binding to a receptor on the host membrane. Many picornaviruses have similar receptor molecules that are members of the immunoglobulin superfamily (IgSF), whose extracellular regions comprise two to five amino-terminal immunoglobulin-like domains. For example, poliovirus receptor (PVR, CD155) contains three amino-terminal domains [4]. Additionally, coxsackievirus B1-B6 receptors (coxsackievirus-adenovirus receptor, CAR) [5], and the receptors for coxsackievirus A21 and major-group human rhinovirus (intercellular adhesion molecule-1, ICAM-1), have two and five amino-terminal domains, respectively [6,7]. In all of these receptors, the amino-terminal domain, D1, is involved in the binding with the conserved amino acid residues of the picornavirus canyon, which can trigger viral instability and uncoating. Therefore, a single receptor suffices for virus entry, especially for poliovirus and rhinovirus [8]. However, some viruses can use non-IgSF cell surface receptors that bind to the outside of the canyon. For example, the low-density lipoprotein receptor (LDL-R) is used by the minor-group of rhinoviruses [9], and the decay-accelerating factor (DAF or CD55) binds to some echoviruses and group B coxsackieviruses [5,10]. Human P-selectin glycoprotein ligand-1 and scavenger receptor B2 are cellular receptor for enterovirus 71 [11,12]. Furthermore, foot-and-mouth disease viruses can attach to integrin α_v_β_3 _or heparin sulfate, as determined by the cell lines and the virus isolates [13,14]. These interactions, however, do not cause viral instability or uncoating, but probably result in the aggregation of other receptors, or trigger the subsequent endocytosis. For example, coxsackievirus B3 (CVB3) recruits CAR to the site of infection by binding to a second receptor DAF that is expressed in the epithelial cells [5]. Also, coxsackievirus A21 binds DAF for entry into cells only in the presence of a coreceptor, ICAM-1 [6]. Indeed, receptor recognition is important in determining the tropism of the cell and host range. However, the interaction of capsid proteins with intracellular host factors is also significant. For example, VP2 of CVB3, but not VP2 of other picornaviruses, may specifically bind to proapoptotic protein Siva, and affect the induction of apoptosis, viral spread, and the pathological process of CVB3-caused disease [15]. As will be discussed below, factors other than virus-receptor interaction, including cellular factors (such as polypyrimidine tract-binding protein, PTB) and viral genome elements (such as internal ribosome entry site, IRES), both interact with the 5' untranslated region (5' UTR) and thus influence the efficiency of translation initiation and virus replication.

### Viral proteolytic activities influence cellular functions

Leader, 2A and 3C proteases (L^pro^, 2A^pro ^and 3C^pro^) are picornavirus-encoded proteases, and are important for viral polyprotein processing [16-18]. L^pro ^is present only in aphtovirus and cardiovirus-infected cells. Viral proteases not only cleave viral polypeptides, but also inhibit various host machineries.

Picornaviral 3C^pro ^can reportedly enter nuclei through its precursor 3CD' or 3CD, which contains a nuclear localization sequence (NLS) [19,20]. 3C^pro ^can cleave numerous factors and regulators that are associated with cellular DNA-dependant RNA polymerase I, II and III, such as TATA-box binding protein (TBP), octamer-binding protein (OCT-1), transcription activator p53, cyclic AMP-responsive element binding protein (CREB), histone H3 and DNA polymerase III [21-26]. 3C^pro ^may be involved in the virus-induced blockage of host transcription. 2A^pro ^reportedly cleaves TBP *in-vitro*, but cannot inhibit cellular transcription [27].

2A^pro ^and L^pro ^cleave eIF4GI and eIF4GII, 3C^pro ^cleaves eIF4AI, which lead to the shut off of host translation [28-32]. The cleavages of Poly(A) binding protein (PABP) by 2A^pro ^and 3C^pro ^also contributes to the inhibition of cellular translation [33,34]. Furthermore, EMCV 2A protein without a catalytic function reportedly associates with 40S ribosome subunit, suggesting another mechanism of host translation shut off [35].

Numerous cytoskeleton-associated factors are cleaved in virus infection. 3C^pro ^cleaves microtubulin-associated protein 4 and 2A^pro ^cleaves cytokeratin 8, which may be associated with the virus-induced cytopathic effect [36,37]. 2A^pro ^of enteroviruses, such as the coxsackievirus B group cleaves dystrophin protein, which may be the factor leading to cardiomyopathy [38-40]. Additionally, 2A^pro ^and 3C^pro ^reportedly induce cell apoptosis via both classic and autophagy pathways [41-44].

Infection by poliovirus and rhinovirus involves accumulation of nuclear proteins in cytoplasm. Several components of the nuclear pore complex were degraded in infected cells and 2A^pro ^has been suggested to be a factor that blocks nucleo-cytoplasmic trafficking [45-48].

### Viral and host proteins involved in RNA replication

Picornavirus infection induces a change in host membrane permeability and the production of membranous structures, on which viral replication depends [49-52]. The viral replication complex has been identified to associate tightly with virus-induced membranous vesicle [53], various replication-associated viral proteins, such as 2B, 2BC, 3A, and 3D [52,54,55] and host proteins (Table 1).

**Table 1:** Cellular proteins involved in picornaviral RNA replication

Proteins	Binding sites/associated viral proteins	Viruses	Refs
PCBP1	Cloverleaf	PV	[93,95]
PCBP2	Cloverleaf, 3CD, 3C	PV	[92,93,106]
hnRNP C	Negative strand 3' stem-loop I, 3D, 3CD, P2, P3	PV	[97]
hnRNP K	5' UTR	EV71	[96]
Sam68	3D	PV, CVB3	[105,169]
PABP	3CD, stem-loop I and poly(A) tail	PV	[106,107]
OCT-1	3CD, 3C	PV, HRV16	[19,23]
B23	3CD	HRV16	[19]
La	3' and 5' UTR	CVB3	[112]
EF-1α	3CD, stem-loop I	PV	[91]

*PCBP, poly(rC) binding protein; hnRNP, heterogeneous nuclear ribonucleoprotein; PABP, poly(A)-binding protein; La, Lupus autoantigen; EF-1α, eukaryotic elongation factor 1 alpha.

### 2B/2BC

Viral protein 2B and its precursor 2BC have been suggested to be responsible for membranous alteration in infected cells [56-60]. The cellular proteins of COPII have reportedly been used in the virus-induced production of vesicles [61]. 2B and the precursor 2BC contain two hydrophobic regions, which are α amphipathic a-helix domain, which is important in multimerization, integrating into the membrane of the host Golgi and ER complex, producing virus-induced vesicles, and forming the virporin complex [60,62-64]. The accumulation of 2B or 2BC proteins on Golgi changes the permeability of plasma membrane [56,62] and the disassembly of Golgi complex [65], causing cell lysis [57]. The membrane that integrates the 2B/2BC complex also reduces the Ca^2+ ^level in ER and Golgi complex by increasing the efflux of Ca^2+ ^[57]. The disruption of Ca^2+ ^homeostasis by 2B/2BC is the mechanism why the transport of protein from ER to Golgi is blocked [58,59,66,67]. The 2B-induced intracellular Ca^2+ ^imbalance is also related to the anti-apoptosis property [68]. Hepatitis A virus 2B protein can reportedly inhibit cellular IFN-β gene transcription by blocking the activation of the interferon regulatory factor 3 (IRF-3), which has been suggested to be crucial to the survival of the virus [69].

### 3A

Protein 3A, a membrane binding protein, plays a role in inhibiting cellular protein secretion and mediating presentation of membrane proteins during viral infection. The expression of poliovirus 3A protein in cells disrupts ER-to-Golgi trafficking, which is also observed in poliovirus 2B-expressing cells [58,65,70]. Moreover, the interference of protein trafficking by 3A is caused by the redistribution of ADP-ribosylation factor (Arf) family, which are important components of the membrane secretion pathway [71]. The cycling of Arf proteins between active GTP-bound and inactive GDP-bound forms are mediated by guanine nucleotide exchange factors (GEFs) and Arf GTPase-activating proteins (GAPs) [72]. Brefeldin A (BFA), a metabolite from fungus, blocks protein trafficking from ER to Golgi in cells by inhibiting the regeneration of Arf-GTP from Arf-GDP [73]. BFA can also inhibit the replication of poliovirus, implying the participation of Arf proteins in viral RNA replication [74-76]. During poliovirus infection, the Arf family is involved in vesicle formation from various intracellular sites through interacting with numerous regulatory and coat proteins, and translocating to the site of viral RNA replication [71]. Two individual viral proteins, 3A and 3CD, can recruit Arfs to bind to membranes via different mechanisms [77,78]. The expression of 3A results in the recruitment of Arfs to membranes by specifically recruiting the cellular GEF, and Golgi-specific brefeldin A resistance factor 1 (GBF1). However, synthesis of 3CD causes other GEFs, Brefeldin A-inhibited guanine nucleotide exchange factor 1 (BIG1) and BIG2, to associate with membranes [77].

### 3AB

According to biochemical data, the 3AB protein is a multifunctional protein. The hydrophobic domain in the 3A portion of the protein associates with membrane vesicles [79,80]. This interaction is believed to anchor the replication complex to the virus-induced vesicles. Recombinant 3AB interacts with poliovirus 3D and 3CD *in vitro *[81]. The membrane-associated 3AB protein binds directly to the polymerase precursor 3CD on the cloverleaf RNA of the poliovirus, stimulating the protease activity of the 3CD, and may serve as an anchor for 3D polymerase in the RNA replication complexes [82]. Adding 3AB stimulated the activity of poliovirus 3D polymerase *in vitro *[83]. Furthermore, 3AB has been demonstrated to function as a substrate for 3D polymerase in VPg uridylylation [84]. The 3AB protein, rather than 3B (the mature VPg), has been proposed to be delivered to the replication complexes for VPg uridylylation. Poliovirus 3AB exhibits other functions, such as helix destabilization, revealing that 3AB has the nucleic acid chaperon activity in destabilizing the secondary structures of RNA and enhancing the hybridization in complementary nucleic acids in viral replication [85].

### 3B

The enteroviral and rhinoviral 3B proteins (VPg) are small peptides, containing 21 to 23 amino acids, which are covalently linked with the 5' termini of picornavirus genome via a 5' tyrosyluridine bond in the conserved tyrosine residue in the VPg. VPg has been shown to interact with poliovirus 3D polymerase, which incorporates UMP in VPg, yielding VPgpU and VPgpUpU [86]. These products are observed in both poliovirus-infected cells and crude replication complex extract [87]. The uridylylated VPg is utilized as a primer in both positive- and negative-strand RNA synthesis [88].

### 3CD

3CD protein, i.e. the precursor of mature 3C protease and 3D polymerase, exhibits protease activity but no polymerase activity [89]. 3CD is capable of processing the poliovirus P1 precursor region [90]. Poliovirus 3CD contributes to viral RNAreplication by circularization of the viral genome via interacting with both 5' and 3' ends of viral RNA [91]. The cellular poly(rC) binding proteins (PCBPs), involved in viral IRES-driven translation, is also identified in the ribonucleoprotein complex, which contains 3CD and have a stem-loop I structure at the 5' end of the poliovirus genome [92,93]. PCBPs contain four isoforms (PCBP 1-4) in mammalian cells, but only PCBP1 and 2 have been found to be involved in enterovirus replication [92,93]. PCBP1 and 2 are KH domains RNA-binding proteins, which are involved in the metabolism of cellular mRNAs in normal cells. PCBP2 binds to both poliovirus stem-loop I and IRES, whereas PCBP1 has a binding affinity only for stem-loop I [94,95]. The addition of recombinant PCBP1 rescues viral RNA replication in PCBP-depleted extracts, but does not rescue viral translation [95]. Another cellular protein, heterogeneous nuclear ribonucleoprotein K (hnRNP K), interacts with stem-loops I-II and IV of the EV71 5' UTR. During EV71 infection, hnRNP K was enriched in the cytoplasm where virus replication occurs, whereas hnRNP K was localized in the nucleus in mock-infected cells. Viral yields were found to be significantly lower in hnRNP K knockdown cells and viral RNA synthesis was delayed in hnRNP K knockdown cells in comparison with negative-control cells treated with small interfering RNA [96]. Moreover, 3CD has been shown, using the pull-down assay, to interact with heterogeneous nuclear ribonucleoprotein C (hnRNP C) [97]. The hnRNP C participates in pre-mRNA processing in normal cells. The mutant form of hnRNP C with the defective activity in protein-protein interaction inhibits the synthesis of viral positive-strand RNA, implying the participation of hnRNP C in RNA replication [97]. The interactions of 3CD with these cellular proteins, PCBP, hnRNP C and the viral protein, 3AB, together with the stem-loop I structure of poliovirus, form important complexes in viral RNA replication [98]. Several other cellular proteins have been reported to interact with 3CD. For example, the eukaryotic elongation factor EF-1α, one such cellular cofactor, can interact with the poliovirus 3CD-stem-loop I complex [91]. The transcription factor OCT-1 and the nucleolar chaperone B23, have also been identified as co-localizing in nuclei with HRV-16 3CD during virus infection [19]. Additionally, the recombinant mature HRV-16 3C can cleave OCT-1 *in vitro*. The mature 3C from the precursor 3CD may play a role in shutting off host cell transcription in nuclei. As well as exhibiting protease activity, 3CD interacts with viral RNA structures, the stem-loop I, 3' UTR and the *cis*-acting replication element (*cre*) motif of poliovirus RNA, which is the template for VPg uridylylation [91-93,99,100].

### 3D

The viral RNA-dependent RNA polymerase 3D is one of the major components of the viral RNA replication complex. The purified poliovirus 3D polymerase from the complex exhibits elongation activity [101]. 3D polymerase can also uridylylate VPg and use VPg-pUpU as a primer during viral RNA replication [86,102]. The polymerase-polymerase interaction of poliovirus has been observed in biochemical and crystal structure studies [81,103,104]. The polymerase oligomerization has been proposed to be responsible for efficient template utilization. The host protein, Sam68, was identified as interacting with poliovirus 3D using a yeast-two hybrid system [105]. This interaction has also been observed in poliovirus-infected cells. Sam68, an RNA binding protein, mediates alternative splicing in cells in response to an extracellular signal. The details of the functions of Sam68 in virus RNA replication need to be demonstrated.

### *cis *elements involved in RNA replication

The RNA secondary structures in the viral genome play important roles in the replication of viral RNA. The *cis *elements contain stem-loop I at the 5' terminus of 5' UTR, 3' UTR and poly(A) tail at the 3' terminus of enterovirus RNA. The circularization of the poliovirus template between the 5' and 3' termini of the viral genome is crucial during the initiation of both positive and negative-strand RNA replication [106,107]. The 3CD and PCBP2 on stem-loop I at the 5' terminus of 5' UTR and poly(A)-binding protein (PABP) and 3CD on the 3' termini of genome are involved in the circularization of RNA genome for initiation of negative-strand RNA synthesis [106]. The enterovirus 3' UTR serves as the initiation point of negative-strand RNA synthesis. The 3CD or 3D has the ability for the interaction with the 3' UTR element [91]. The binding of 3CD and 3AB to 3' UTR does not depend on the interaction with host proteins and suffices for viral RNA replication *in vitro *[91]. Moreover, many works have reported that host proteins can bind to the 3' UTR of rhinovirus and enterovirus [91,108,109]. Nucleolin, a nuclear factor, which accumulated in the cytoplasm of poliovirus-infected cells, interacted strongly with an intact 3'-UTR of poliovirus *in vitro *[110]. The immunodepletion of nucleolin from cell-free extract reduced virus reproduction, indicating that nucleolin may be involved in viral RNA replication. The 3' stem-loop I of the negative-strand RNA is the initiation site of positive-strand RNA synthesis. Positive-strand RNA synthesis is initiated by the recruitment of uridylylated VPg-containing replication complexes close to the 3' stem-loop I of the negative strand. Viral protein 2C has been reported to interact directly with the 3' stem-loop I of the negative strand [111]. The cellular protein, hnRNP C, which specifically interacts with either the 3' end of the poliovirus negative-strand RNA or the protein 3CD, is involved in positive-strand RNA synthesis, and probably in the initiation step [97]. Some cellular proteins, such as La, can interact with both 3' and 5' UTRs of CVB3 independently of the poly(A) tail, and may play a role in mediating cross-talk between the 5' and 3' ends of CVB3 RNA for viral RNA replication [112].

Synthesis of the uridylylated VPg is the first step of viral RNA synthesis. The efficient uridylylation of VPg requires 3D polymerase, 3CD protein, UTP and the *cre *motif from the viral genome as the template. 3CD has been shown to stimulate *cre*-mediated VPg uridylylation [102]. Moreover, the 3AB protein is regarded as the precursor of VPg (3B) in the RNA replication complex for VPg uridylylation [84]. The *cre *motif was identified in different regions of the enterovirus genome. The *cre *structures are located in the 2C-encoding region of poliovirus, the capsid-encoding region of human rhinovirus 14 and cardiovirus, the 2A-encoding region of human rhinovirus 2, the 5' non-coding region of the foot-and-mouth disease virus, and the 3D-encoding region of the hepatitis A virus [113-118].

### Switch from translation to RNA replication

The positive-stranded RNA viruses use the same RNA as a template for translation and replication. Ribosomes move from the 5' end to the 3' end of RNA to undergo translation, and RNA polymerase binds to the 3' end of the same RNA to initiate replication. *In vitro *experiments have demonstrated that using cycloheximide to freeze ribosomes on translated RNAs inhibits RNA replication, while using puromycin to release the ribosomes allows facilitates RNA replication [119]. These two events cannot occur at the same time, so the balance between translation and replication is important.

The poliovirus genome contains a conserved 5' UTR, which is important to translation and RNA replication [120]. Gamarnik and Andino have suggested that the binding of 3CD to the cloverleaf at the 5' end of the viral genome promotes the replication of RNA, rather than its translation [121]. Furthermore, they found that PCBP2 binds to stem-loop IV in poliovirus translation. When newly translated 3CD binds to stem-loop I RNA, the affinity of PCBP2 binding for the same region is increased. 3CD induces the dissociation of PCBP2 from stem-loop IV because the affinity of PCBP2 for stem-loop I is increased, while that for viral translation is reduced [98].

Semler *et al*. found that proteases 3C/3CD cleave PCBP1 and 2 during the mid-to-late phase of poliovirus infection. The primary cleavage site is between the KH2 and KH3 domains. The cleaved PCBP2 cannot bind to stem-loop IV and it loses functionality in translation. However, the cleaved PCBP2 still binds to stem-loop I and mediates the replication of viral RNA. PCBP2 can mediate the switch from viral translation to RNA replication [122].

### Host factors and viral proteins involved in picornaviral IRES-mediated translation

**Figure 2:**
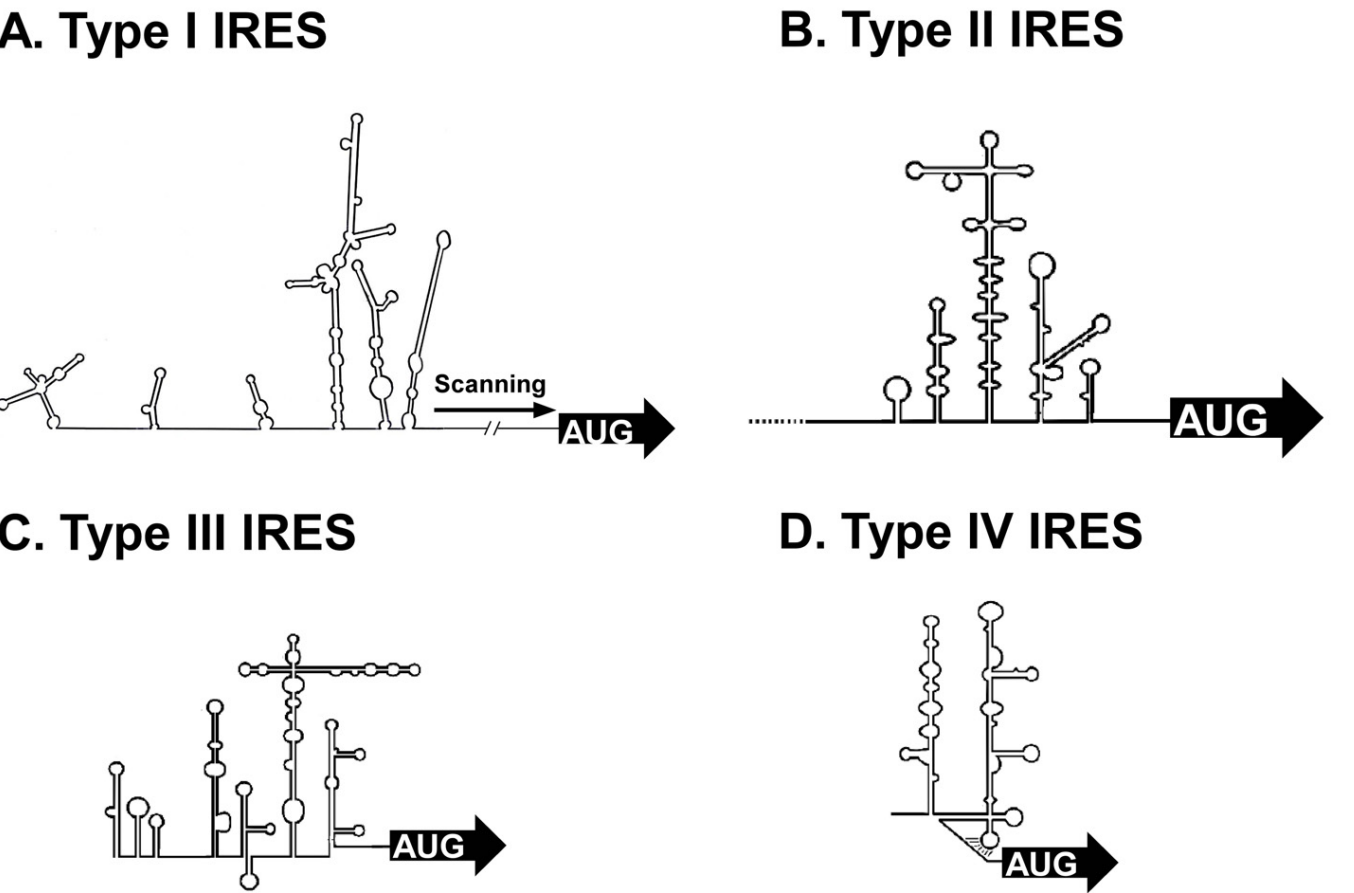
**Structural features of type I, II, III, and IV picornavirus IRES elements**. RNA secondary structures of four types base on M-fold software. (A). Enteroviruses (PV, CVB3 and EV71) and rhinoviruses contain type I IRES elements. (B). Aphthoviruses (FMDV) and cardiovirus (EMCV and TMEV) contain type II IRES elements. (C). HAV contains type III IRES element. (D). porcine reschovirus serotype 1 (PTV-1) contains type IV IRES element.

Most picornaviral IRESs are divided into four classifications based on homology, secondary structure, and other properties (Fig. 2). Type I IRESs include those of poliovirus, rhinovirus, coxsackievirus, and other enteroviruses. Type II IRESs include those of the foot and mouth disease virus (FMDV), cardiviruses such as encephalomyocarditis virus (EMCV), paraechoviruses and kobuvirus. Type III IRESs include the hepatitis A virus (HAV). The newly classified teschovirus IRES is the most similar to hepatitis C virus, which is not a picornavirus, and so may represent an ancient recombinant event between an enterovirus and hepacivirus or pestivirus [123].

IRES-mediated initiation may require both canonical initiation factors and IRES trans-activating factors (ITAFs) that are not involved in cap-mediated initiation. ITAFs are cellular proteins that are not involved in normal cap-dependent translation but facilitate cap-independent translation. Different viral IRESs have different ITAF requirements, although several are shared. ITAFs may serve as IRES chaperones, binding to RNA across multiple domains and stabilizing the entire IRES in a configuration that is appropriate for binding canonical translation factors, and ultimately ribosomes (Table 2) [124].

**Table 2:** Cellular proteins involved in picornaviral IRES-mediated translation

Proteins	Binding sites/associated viral proteins	Viruses	Refs
PTB	IRES, 3C	PV, HRV, EMCV, FMDV, TMEV, HAV	[130-136]
nPTB	IRES	PV, TMEV	[135,143]
PCBP1	Cloverleaf, IRES	PV, HRV	[93,153]
PCBP2	Cloverleaf, IRES, 2A, 3CD, 3C	PV, HRV, HAV, CVB3	[94,152-154,156,170]
Unr	IRES	PV, HRV	[139,157]
La	IRES, 3C	PV, HRV, HAV, CVB3, EMCV	[147,148,150,151,171]
ITAF45	IRES	FMDV	[124]
hnRNP A1	IRES	HRV2, EV71	[158,159]
Nucleolin/C23	IRES	PV, HRV	[162]
DRBP 76: NF45 heterodimer	IRES	HRV2	[163,164]
GAPDH	IRES	HAV	[137,172]
FBP2	IRES	EV71	[168]
PABP	IRES	PV, EMCV	[125,173]

*PTB, polypyrimidine tract-binding protein; nPTB, neural polypyrimidine tract-binding protein; PCBP, poly(rC) binding protein; Unr, upstream element binding protein; La, Lupus autoantigen; hnRNP, heterogeneous nuclear ribonucleoprotein; DRBP 76: NF45 heterdimer, dsRNA binding protein 76: NF45 heterdimer; GAPDH, glyceraldehyde 3-phosphate dehydrogenase; FBP2, far upstream element binding protein 2; PABP, poly(A)-binding protein.

### Canonical translation factors

PV and EMCV IRES elements are similar to capped mRNA in requiring that some initiation factors bind to primed 40S subunits and to the RNA itself. These canonical factors have been identified as eIF4G and eIF4B, which bind to the viral RNA, and eIF3 and eIF2, which must prebind the 40S subunit. Furthermore, poly(A)-binding protein substantially promotes the IRES-mediated translation of EMCV and PV. This rather high requirement of picornaviral IRESs for canonical translation factors is in contrast to that of IRES elements of other viruses, such as hepatitis C virus and the cricket paralysis virus, which require few or no translation factor to bind to the ribosome [125].

### Noncanonical translation factors

#### Polypyrimidine tract-binding protein (PTB)

PTB (also known as p57 and hnRNP I) is a member of the hnRNP family and shuttles between the nucleus and the cytoplasm in a transcription-sensitive manner [126]. PTB is a 57 kDa mRNA splicing factor and has four RNA recognition motifs (RRMs). PTB was identified originally as a protein that binds to the polypyrimidine tracts (Py tracts) of adenoviral major-late and α-tropomyosin pre-mRNAs, and has been proposed to be a splicing factor [127]. The binding of PTB to the Py tract close to the branching point of intron has been demonstrated to modulate the alternative splicing of certain pre-mRNAs [128]. Independently, PTB was shown to interact specifically with the IRESs of numerous picornaviruses [129,130], including poliovirus [131,132], FMDV [133], EMCV [134], Theiler's murine encephalomyelitis virus (TMEV) [135], and HAV [136,137].

Experiments on the depletion and repletion of PTB from rabbit reticulocyte lysate (RRL) have revealed that the efficient translation of FMDV mRNA and a mutant EMCV mRNA depends on PTB [133,134]. Intriguingly, PTB is required for the translation of a mutant EMCV mRNA, but not wild-type EMCV [138], suggesting that PTB is involved in maintaining the proper conformation of the mutant EMCV IRES [138]. Supplementation of RRL with PTB enhances the translation of polioviral mRNA [139,140]. The immunodepletion of PTB from HeLa cell lysate inhibited poliovirus IRES-dependent translation. Repletion of purified PTB to the immunodepleted lysate did not restore poliovirus IRES-dependent translation. This investigation suggested that the depletion process may have removed unidentified translation factors [132]. The effect of PTB on poliovirus IRES-dependent translation was examined using artificial dicistronic mRNAs that contain the PTB gene as the first cistron, the poliovirus IRES in the intercistronic region, and the chloramphenicol acetyltransferase (CAT) reporter gene as the second cistron. When PTB was transfected into HeLa cells, which contain a limited amount of endogenous PTB, its additional expression increased the activity of poliovirus IRES by 2.5-fold [141]. These results suggest that PTB is required for, or at least promotes, the IRES activity of picornavirus.

Poliovirus protein, 3C^pro ^(and/or 3CD^pro^), cleaves PTB isoforms (PTB1, PTB2, and PTB4). PTBs contain three 3C^pro ^target sites (one major target site and two minor target sites). PTB fragments that are produced by PV infection are redistributed to the cytoplasm from the nucleus, where most of the intact PTBs are localized. Additionally, these PTB fragments inhibit poliovirus IRES-dependent translation in a cell-based assay system. The authors posit that the proteolytic cleavage of PTBs may contribute to the molecular switching from translation to the replication of polioviral RNA [142].

PV-attenuated type 3 Sabin and virulent type 3 Leon viruses translated equally well in HeLa cells, but the translation of the attenuated Sabin virus is restricted in neuroblastoma cells. The C472-to-U mutation in the IRES caused the translation defect. Comparison of IRES between Leon serotype 3 and Sabin serotype 3 PV revealed that PTB and a novel neural-cell-specific homologue of PTB (nPTB) were bound adjacent to the attenuation mutation in domain V, but binding was less efficient on the Sabin IRES. The Sabin IRES was demonstrated to have a translation defect in chicken neurons that can be rescued by increased PTB expression in the CNS [143].

PTB has also been shown most clearly to function as an RNA chaperon, stabilizing the type II IRESs, such as EMCV and FMDV, in an active conformation [144,145].

### Lupus autoantigen (La)

Lupus autoantigen (La) is a 52 kDa, predominantly nuclear protein. It is a target for autoimmune recognition in patients with systemic lupus erythematosus and Sjogren's syndrome. The majority of La is localized in the nucleus, and it is related to stabilization of nascent RNAs, nuclear retention of nascent transcripts, and RNA pol III transcription termination. During PV infection, La is redistributed to the cytoplasm by 3 h postinfection (p.i.) [146,147]. This redistribution coincides with the appearance of 3C^pro ^in infected cells, and is caused by a 3C^pro^-mediated cleavage event, which removes a nuclear localization signal from the C-terminus of La, but maintains the dimerization domain. The truncated La can still stimulate translation effectively, but is relocalized to the cytoplasm during viral protein synthesis [147]. Depletion of La from cells by small interfering RNA reduced IRES translation. Similarity, a dominant-negative mutant of La inhibited 40S ribosomal binding by PV IRES *in vitro *[148]. La protein also binds to the CVB3 IRES and stimulates viral translation in a dose-dependent manner in rabbit reticulocyte [149,150]. La protein binds specifically to distinct parts of HAV IRES, and suppresses HAV IRES-mediated translation and replication by small interfering RNA *in vivo *and purified La protein *in vitro *[151].

### Poly(rC)-binding protein 1, 2 (PCBP1, 2)

PCBPs are RNA-binding proteins that preferentially bind to single-stranded stretches of cytidines. Mammalian cells contain four isoforms, PCBP 1-4, but only PCBP1 and PCBP2 have been experimentally demonstrated to have roles in the life cycles of enteroviruses [93]. PCBP2 is a factor that is required for poliovirus translation and was discovered because of its interaction with stem-loop IV of the poliovirus IRES [94]. Depletion and replication studies of PCBP2 from HeLa cell-free extracts using a stem-loop RNA affinity column have revealed that PCBP2 was required for PV IRES-dependent translation [152]. Moreover, an oligo-DNA with high affinity to PCBP1 and PCBP2 was recently used to prove that both PCBP1 and PCBP2 function as ITAFs of the PV IRES [153]. PCBP2 also binds to the HAV 5' UTR and stimulates viral translation [154].

One nucleocytoplasmic SR protein, SRp20, interacts with PCBP2 and is involved in the internal ribosome entry site-mediated translation of viral RNA. Both depletion and *in vitro *translation studies of SRp20 from HeLa cell free extracts have shown that SRp20 is required for the initiation of PV translation. Targeting SRp20 in HeLa cells with short interfering RNAs inhibited the expression of SRp20 protein and correspondingly reduced PV translation [155].

*In vitro *translation reactions were performed in HeLa cell cytoplasmic translation extracts whose cellular protein, PCBP2 was depleted [156]. Upon depletion of PCBP2, these extracts exhibited a significantly reduced capacity to translate reporter RNAs that contained the type I IRES elements of poliovirus, coxsackievirus, or human rhinovirus, which is linked to luciferase; however, adding recombinant PCBP2 protein restored translation. RNA electrophoretic mobility-shift analysis demonstrated specific interactions between PCBP2 and both type I and type II picornavirus IRES elements; however, the translation of reporter RNAs that contain the type II IRES elements of the encephalomyocarditis virus and the foot-and-mouth disease virus did not depend on PCBP2. These data indicate that PCBP2 is essential for the internal initiation of translation on picornavirus type I IRES elements, but not by the structurally distinct type II elements [156].

### Upstream of N-ras (Unr)

Upstream of N-ras (Unr) is a cytoplasmic protein that contains five cold-shock domains. A depletion study of Unr from reticulocyte lysate revealed that Unr was required for the translation of rhinovirus IRES [139]. Both HRV and PV IRES translation was severely impaired in unr(-/-) murine embryonic stem cells. Translation was restored by the transient expression of Unr in unr(-/-) cells [157].

### Heterogenerous nuclear ribonucleoprotein A1 (hnRNP A1)

hnRNP A1, an RNA-binding protein that shuttles between the nucleus and the cytoplasm, is a member of a large group of RNA binding proteins (hnRNPs) which are classified into several families and subfamilies based on conserved structural and functional motifs. The hnRNP A1 protein is composed of 320 amino acids; it contains two RNA-binding domains and a glycine-rich domain, which is responsible for protein-protein interaction.

hnRNP A1 is an internal ribosome entry site (IRES) *trans*-acting factor that binds specifically to the 5' UTR of the HRV2 and regulates its translation. Furthermore, the cytoplasmic redistribution of hnRNP A1 after rhinovirus infection enhances rhinovirus IRES-mediated translation [158]. RNA-protein pull down assay, reporter assay and viral RNA synthesis assay reveal that hnRNP A1 also interacts with the 5' UTR of enterovirus 71 (EV71) and regulates viral replication [159].

### ITAF45

ITAF45 is also known as erbB3-binding protein 1 (Ebp1) or p38-2G4, which is a proliferation-dependent protein that is distributed throughout the cytoplasm from the metaphase to the telophase. ITAF45 is a proliferation-dependent protein that is undetectable in murine brain cells and so may function as a tissue-specific factor that controls the translation of particular mRNAs. The initiation on the TMEV IRES depended strongly on PTB, where the initiation on the FMDV IRES depended on both PTB and ITAF45. ITAF45 was bound specifically to a central domain of the FMDV IRES and acted synergistically with PTB to promote the binding of eIF4F to an adjacent domain [124].

### Nucleolin/C23

As a 110 kDa nucleolar protein, nucleolin/C23 protein is also an RNA binding protein which contains four RNA binding motifs [160]. Nucleolin is an abundant protein of the nucleolus and participates in rDNA transcription, rRNA maturation, ribosome assembly and nucleocytoplasmic transport [161]. Nucleolin/C23 has been shown to translocate into the cytoplasm following the infection of cells with PV [110]. Nucleolin/C23 stimulates PV IRES-mediated translation *in vitro *and rhinovirus IRES-mediated translation *in vivo*. Nucleolin/C23 mutants that contain the carboxy-terminal RNA binding domains, but lack the amino-terminal domains, act as dominant-negative mutants in *in vitro *translation assay. The translation inhibitory activity of these mutants is related to their capacity to bind to the 5' UTR sequence [162].

### dsRNA binding protein76:NF45 heterodimer

The double-stranded RNA binding protein 76 (DRBP76) contains two dsRNA-binding motifs and is almost identical to M-phase phosphoprotein 4, NF90, translation control protein (TCP80), and NF associated with dsRNA. DRBP76 has been found to bind to the HRV2 IRES in neuronal cells and to inhibit PV-RIPO translation and propagation [163]. The size of exclusion chromatography indicates that DRBP76 heterodimerizes with the nuclear factor of activated T cells, of size 45 kDa (NF45), in neuronal but not in glioma cells. The DRBP76:NF45 heterodimer binds to the HRV2 IRES in neuronal but not in glioma cells. Ribosomal profile analyses have demonstrated that the heterodimer preferentially associates with the translation apparatus in neuronal cells, and arrests translation at the HRV2 IRES, preventing the assembly PV-RIPO RNA into the polysome [164].

### Far upstream element binding protein 2 (FBP2)

The far upstream element binding protein 2 (FBP2) is also known as the K homology (KH)-type splicing regulatory protein (KSRP). It was originally identified as a component of a protein complex that assembles on an intronic c-src neuronal-specific splicing enhancer, and as an important adenosine-uridine element binding protein (ARE-BP) that interacts with several AREs [165,166]. FBP2 is required for the rapid decay of several ARE-containing mRNAs both *in vitro *and *in vivo*. It contains four contiguous KH motifs that recognize the ARE, interact with the exosome, and the poly(A) ribonuclease (PARN), and promote the rapid decay of ARE-containing RNAs [167].

Biotinylated RNA-affinity chromatography and proteomic approaches were utilized to identify FBP2 as an ITAF for EV71. During EV71 infection, FBP2 was enriched in cytoplasm where viral replication occurs, whereas FBP2 was localized in the nucleus in mock-infected cells. The synthesis of viral proteins was promoted in FBP2-knockdown cells that were infected by EV71. IRES activity in FBP2-knockdown cells exceeded that in the negative control (NC) siRNA-treated cells. However, IRES activity decreased when FBP2 was over-expressed in the cells. The results of this investigation suggest that FBP2 is a novel ITAF that interacts with EV71 IRES and negatively regulates viral translation [168].

Compelling evidence suggests that cellular RNA-binding proteins (ITAFs) are involved importantly in translation from a variety of IRES elements, suggesting potential roles for RNA-binding proteins in IRES-dependent translation. First, an RNA-binding protein recruits the translational machinery via a protein-protein or protein-RNA interaction. This putative RNA-binding protein then binds directly to the ribosomal subunit, to canonical translation factors, or to a putative mediator protein that connects other RNA-binding proteins with the basal translational machinery. Second, RNA-binding proteins may serve as 'clamping proteins,' holding various parts of IRES RNA in a particular configuration. Components of the translational machinery may bind exclusively to the RNA portion of the RNA-protein complex that is maintained by these clamping proteins.

## Concluding remarks

Picornaviruses use multiple RNA-protein interactions to mediate important reactions in their life cycle, including IRES-mediated translation, possible circularization of the genome, and RNA replication. The molecular mechanisms by which host factors are involved in RNA-protein/protein-protein interaction have been intensively studied; however, tissue-specific viral virulence remains unclear, and demands further investigation in the future. No information is available on whether picornaviruses can be targeted by cellular microRNAs, leading to transcriptional or translational silencing, or RNAi-mediated degradation of viral RNA. This is another field that needs to be further studied in the future.

## Competing interests

The authors declare that they have no competing interests.

## Authors' contributions

JYL: Contribution of the part of the induction of background, host factors and viral proteins involved in IRES-mediated translation and conclusion. SCC: Contribution of the part of host factors and capsid proteins that are involved in receptor binding. TCC: Contribution of the part of viral and host proteins involved in RNA replication. KFW: Contribution of the part of viral proteolytic activities influence cellular functions and viral and host proteins involved in RNA replication (2B/2BC). LLC: Contribution of the part of switch from translation to RNA replication. SRS: Contribution of a deep revision of the full text. All authors have read and approved the final manuscript.
